# Blood-Based Markers of Neuronal Injury in Adult-Onset Myotonic Dystrophy Type 1

**DOI:** 10.3389/fneur.2021.791065

**Published:** 2022-01-20

**Authors:** Ellen van der Plas, Jeffrey D. Long, Timothy R. Koscik, Vincent Magnotta, Darren G. Monckton, Sarah A. Cumming, Amy C. Gottschalk, Marco Hefti, Laurie Gutmann, Peggy C. Nopoulos

**Affiliations:** ^1^Department of Psychiatry, University of Iowa Hospital and Clinics, Iowa City, IA, United States; ^2^Department of Radiology, University of Iowa, Iowa City, IA, United States; ^3^Institute of Molecular, Cell and Systems Biology, University of Glasgow, Glasgow, United Kingdom; ^4^Department of Pathology, University of Iowa Hospital and Clinics, Iowa City, IA, United States; ^5^Department of Neurology, Indiana University School of Medicine, Indianapolis, IN, United States

**Keywords:** NF-L protein, myotonic dystrophy 1, central nervous system, diffusion magnetic resonance imaging, tau proteins

## Abstract

**Introduction:**

The present study had four aims. First, neuronal injury markers, including neurofilament light (NF-L), total tau, glial fibrillary acidic protein (GFAP) and ubiquitin C-terminal hydrolase (UCH-L1), were compared between individuals with and without adult-onset myotonic dystrophy type 1 (DM1). Second, the impact of age and CTG repeat on brain injury markers was evaluated. Third, change in brain injury markers across the study period was quantified. Fourth, associations between brain injury markers and cerebral white matter (WM) fractional anisotropy (FA) were identified.

**Methods:**

Yearly assessments, encompassing blood draws and diffusion tensor imaging on a 3T scanner, were conducted on three occasions. Neuronal injury markers were quantified using single molecule array (Simoa).

**Results:**

The sample included 53 patients and 70 controls. NF-L was higher in DM1 patients than controls, with individuals in the premanifest phases of DM1 (PreDM1) exhibiting intermediate levels ($\chi_{\left(2\right)}^{2}=$38.142, *P* < 0.001). Total tau was lower in DM1 patients than controls (Estimate = −0.62, 95% confidence interval [CI] −0.95: −0.28, *P* < 0.001), while GFAP was elevated in PreDM1 only (Estimate = 30.37, 95% CI 10.56:50.19, *P* = 0.003). Plasma concentrations of UCH-L1 did not differ between groups. The age by CTG interaction predicted NF-L: patients with higher estimated progenitor allelege length (ePAL) had higher NF-L at a younger age, relative to patients with lower CTG repeat; however, the latter exhibited faster age-related change (Estimate = −0.0021, 95% CI −0.0042: −0.0001, *P* = 0.045). None of the markers changed substantially over the study period. Finally, cerebral WM FA was significantly associated with NF-L (Estimate = −42.86, 95% CI −82.70: −3.02, *P* = 0.035).

**Interpretation:**

While NF-L appears sensitive to disease onset and severity, its utility as a marker of progression remains to be determined. The tau assay may have low sensitivity to tau pathology associated with DM1.

## Introduction

Myotonic dystrophy type 1 (DM1) is an autosomal dominant, multisystemic disease caused by an expansion of a CTG trinucleotide repeat in the 3′ untranslated region of the *dystrophia myotonica* protein kinase gene (*DMPK*; OMIM 160900). Abnormalities in white matter—as measured with fractional anisotropy (FA)—are one of the most replicated findings in adult-onset DM1 (1–8) and appear to be a global phenomenon rather than tract specific (9). For instance, our group showed that relative to controls, DM1 patients exhibited reduced FA and increased axial diffusivity and radial diffusivity across tracts (9). Alterations in white matter (WM) appear to occur early in the disease, as cerebral WM FA was found to be reduced among individuals with PreDM1, who have yet to manifest clinical motor symptoms (10). Reduced WM FA is associated with motor symptoms of DM1 (9), and with other functional outcomes, such as lower IQ, apathy and hypersomnolence (11). These studies underscore the importance of tracking brain health in DM1 to gain insights into disease onset and progression. Examination of a variety of relevant markers of brain injury could be beneficial in identifying outcome measures for clinical trials. Protein markers of brain injury have garnered considerable attention in the past several years as potential biomarkers of neurodegenerative illness (12–15); however, their utility in tracking disease onset and progression in DM1 is largely unexplored.

Two studies have evaluated brain injury markers in cerebrospinal fluid (CSF) samples of DM1 patients, with both reporting on tau protein (16, 17). Total tau was found to be elevated among DM1 patients, and associations between elevated tau and neurocognitive difficulties (16) and CNS ventricular widening were noted (17). Beside tau, there has been no exploration of brain injury markers in DM1, highlighting a gap in the literature.

Neurofilament light chain (NF-L) is a cytoskeletal protein that is abundantly expressed in myelinated axons (12). Research in Huntington's disease (HD) provides relevant examples of the NF-L's utility as a prognostic marker of neurodegeneration, with one study showing that NF-L concentrations increased with advancing disease (15). Other relevant markers of neurodegenerative disorder are glial fibrillary acidic protein (GFAP) and ubiquitin C-terminal hydrolase-L1 (UCH-L1) (18, 19). GFAP is a marker of astrocytic activity, and UCH-L1 is located mainly in the neuronal cell body. Evaluations of a range of markers of brain injury will broaden the scope and depth of our understanding of pathological processes leading to CNS morbidity in DM1. Moreover, these markers can now reliably be quantified from blood (15), which is more accessible than CSF.

The Iowa DM1 Brain and Muscle Study was designed to evaluate CNS phenotypes of adult-onset DM1 in a prospective manner. The present study had four aims. First, we compared concentrations of plasma markers of injury (NF-L, total tau, GFAP, and UCH-L1) between controls, PreDM1, and manifest DM1. We hypothesized that the manifest DM1 group would exhibit higher plasma concentrations of these markers relative to controls, indicating increased CNS morbidity. The PreDM1 group was expected to exhibit intermediate levels of brain injury markers. Second, we determined the impact of CTG repeat on age-related change in brain injury markers among patients, where longer repeats were expected to be associated with accelerated age-related change in brain injury markers compared with shorter repeats. Third, we evaluated if changes in brain injury markers could be detected over a 3-year study period. We hypothesized that plasma marker concentrations would increase significantly in DM1 patients as a result of disease progression. Fourth, we determined associations between brain injury markers and cerebral WM FA, where increased concentrations of plasma markers were expected to be associated with increased WM morbidity (i.e., reduced WM FA).

## Materials and Methods

### Participants

Individuals with DM1 were recruited to the University of Iowa through the advocacy group The Myotonic Dystrophy Foundation, and by word of mouth. Control participants were recruited from the Iowa City area via advertisements, or as a spouse/partner of patients. Exclusion criteria for all participants included: MRI contraindication, learning disability, a history of serious head injury, or a chronic neurological disorder other than DM1. Control participants were additionally required to be without history of substance abuse, psychiatric illness, or major medical disease.

**Figure 1:**
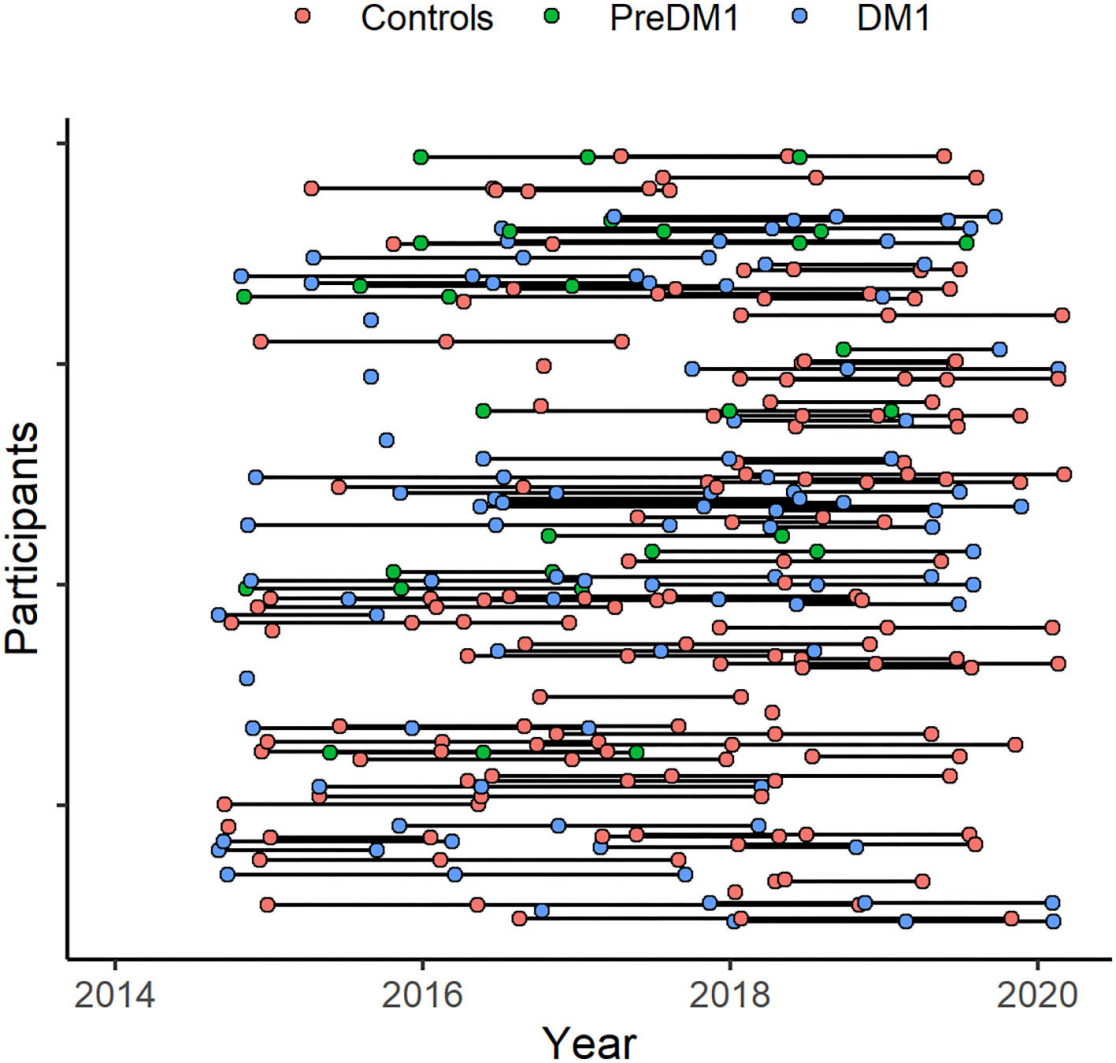
Participant assessment over the study period. Assessments took place between 2014 and 2020 (*x*-axis). Participants (*y*-axis) were assessed on multiple occasions, approximately 1 year apart. The timing of baseline assessments varied across participants and not everyone completed all planned assessments.

Recruitment was targeted to adult-onset DM1 only (onset after age 18). We enrolled 62 controls, 48 patients who had been genetically confirmed to carry the causative mutation expansion in the *DMPK* gene (CTG≥50), and 13 individuals with a family history of DM1 who themselves had not undergone confirmative testing (i.e., ‘at-risk'). Participants underwent genetic testing for research purposes only. At-risk individuals who were determined to have CTG repeat length ≥ 50, were included in the DM1 group (*N* = 5); the remainder were included in the control group (*N* = 8). Assessments took place between September 2014 and March 2020. Participants completed the same assessments on three occasions, approximately 1 year apart (Figure 1). Not all participants completed all three assessments. We utilized mixed modeling to accommodate variation in frequency of follow-up.

PreDM1 was operationally defined by the absence of detectable motor symptoms as determined through clinical examination by a neuromuscular neurologist (LG) using the Muscle Impairment Rating Scale (MIRS). The MIRS ranges from 1 (no symptoms) to 5 (severe symptoms) (10). Of 53 DM1 subjects, 13 were determined to be PreDM1.

### Standard Protocol Approvals, Registrations, and Patient Consents

All data were de-identified, and participants consented to non-disclosure of genetic results. Participants gave written, informed consent prior to enrolling in the protocol. The study was approved by the University of Iowa Institutional Review Board.

### Quantification of Plasma Markers of Brain Injury

Plasma samples were frozen at −80°C, then shipped to Quanterix Inc. for analysis. Concentrations of NF-L, total tau, GFAP and UCH-L1 were quantified using the Simoa Human Neurology 4-Plex A assay (N4PA) (20). The biomarker values in all samples were within the linear range of the assays. Three control observations for UCH-L1 fell below the analytical lower limit of quantification and were removed from the analyses.

### DMPK CTG Repeat Genotyping

Genotyping of the expanded CTG repeat in DM1 mutation carriers was determined using small pool-PCR (SP-PCR) (21, 22). For each patient, four reactions were completed, each using 300 pg genomic DNA template derived from blood leukocytes. CTG repeat lengths were estimated by comparison against DNA fragments of known length and molecular weight markers, using CLIQS software (TotalLabs UK Ltd.). The lower boundary of the expanded molecules in SP-PCR was used to estimate the progenitor (inherited) allele length (ePAL) (23). ePAL is the major determinant of age at symptom onset and disease severity in DM1 (23, 24).

For control individuals, the length of the CTG repeat length was determined by MiSeq sequencing, essentially as described for HD alleles (25), where HD-specific primers were substituted for DM1-specific primers, using reference sequences comprising 0 to 100 CTG repeats and DM1 5′- and 3′-flanking sequences.

### MRI Acquisition and Processing

Participants who took part prior to June 2016 (25% of the sample) were scanned using a 3T Siemens TrioTIM scanner. Those who participated after June 2016 were scanned using a 3T General Electric Discovery MR750w scanner. Batch effects in diffusion-weighted images associated with scanner versions were normalized using ComBat harmonization (Supplementary Figure 1) (26). Diffusion-weighted images were collected using echo planar recovery magnitude sequences collected in the axial plane with either a single-shell (B1000, 64 directions), multi-shell (B1000 and B2000, 29–30 directions per shell), or both. Details regarding image processing of the diffusion-weighted images were previously published (10). Since WM abnormalities in DM1 appear to be global rather than tract-specific (9, 10), we limited our analyses to cerebral WM FA consisting of all supratentorial WM. Note that tract-based comparisons of WM across groups in this sample were reported previously (9).

### Statistical Approach

All analyses were performed in a blinded manner. The distributions of the outcome variables were inspected using Q-Q plots (Supplementary Figure 2). Across aims, NF-L, total tau, GFAP and UCH-L1 were included as dependent variables in separate models. Models were compared using Akaike information criteria with a correction for small sample sizes (AICc), and models with the smallest AICc were selected. Mixed multivariable linear models were conducted, which included random effects for participants to account for non-independency of the data. Random intercepts and slopes were added as specified for each aim below. All model comparisons are listed in the Supplementary Materials.

The first aim was to compare brain injury markers across groups; hence, the group predictor (controls vs. PreDM1 and controls vs. DM1) was the variable of interest. Random intercepts and slopes for age at evaluation were added to account for the impact of age. Models that were compared included a combination of the following predictors: group, age at assessment, sex, and the interaction between group and age at evaluation (Supplementary Tables 1–4). The omnibus effect of group was evaluated prior to interpreting differences across group levels.

The next set of analyses were limited to DM1 groups only. The second aim was to evaluate the impact of ePAL on brain injury markers in DM1 patients. In addition to the variable of interest (ePAL), models included age at evaluation (linear and quadratic polynomial terms), sex, and the interaction between age and ePAL (Supplementary Tables 5–8). Random intercepts and slopes were added for age at assessment.

Years on study was the predictor of interest for aim 3 since its goal was to identify changes in brain injury markers. Years on study, or elapsed time, was taken as the number of days between visits, which was converted to year units. All models included random intercepts and slopes for age at baseline. Sex, group (PreDM1 vs. DM1), and age at baseline were additionally considered as main effects for the purpose of model comparisons, as well as a group by years on study, and group by age at baseline interaction effects (Supplementary Tables 9–12). Mixed linear models allow for estimates of change in the presence of random missing observations.

The fourth aim was to determine associations between brain injury markers and WM FA in DM1 patients, with WM FA being the predictor variable of interest. Random intercepts and slopes for age at assessment were added. Age at evaluation, sex, and group (PreDM1 vs. DM1) were also considered, in addition to interactions between WM FA by age at assessment, group by age interaction (Supplementary Tables 13–16).

## Results

### Sample

The sample included 70 controls, 13 individuals with PreDM1, and 40 patients with manifest DM1 (Table 1). Across the entire sample, 123 individuals completed one visit (41.41%), 107 (36.03%) completed two visits, and 67 (22.56%) completed three visits. There were no significant differences in the distribution of number of visits across the three groups ($\chi_{\left(4\right)}^{2}=$1.18, *P* = 0.882; Table 2). On average, 0.98 years had elapsed between visits, which was similar across groups: *F*_(2)_ = 1.54, *P* = 0.219. The average age of the sample at baseline was 44.8 years old SD = 12.2, with no significant differences in mean age between groups [*F*_(2)_ = 0.84, *P* = 0.43]. While there were significantly fewer men (35%) than women (65%) $\chi_{\left(1\right)}^{2}=$11.13, *P* < 0.001; the distribution of sex was similar across groups $\chi_{\left(2\right)}^{2}=$1.17, *P* = 0.558. The PreDM1 group had a shorter ePAL than did the manifest DM1 group (Estimate = 78.77, SD = 28.56, *t*_(50)_ = 2.76, *P* = 0.008). Average disease duration, determined through self-reported onset of motor or muscle symptoms, was 12.9 years (SD = 7.29 years; Table 1). Supplementary Table 17 summarizes descriptive statistics across groups and visits.

**Table 1:** Sample demographics.

	**Controls**	**PreDM1**	**DM1**
	**(*N =* 70)**	**(*N =* 13)**	**(*N =* 40)**
**Age (years)**			
Mean (SD)	43.6 (12.8)	47.4 (16.3)	46.0 (9.42)
Median [Min, Max]	43.7 [18.3, 63.4]	53.3 [19.2, 64.0]	46.1 [30.3, 62.2]
**Sex**			
Female	45 (64.3%)	7 (53.8%)	28 (70.0%)
Male	25 (35.7%)	6 (46.2%)	12 (30.0%)
**Disease duration**			
Mean (SD)	–	–	12.9 (7.29)
Median [Min, Max]	–	–	12.8 [2.42, 28.9]
Missing	–	–	3 (7.5%)
**MIRS**			
1	7 (10.0%)	13 (100%)	0 (0%)
2	1 (1.4%)	0 (0%)	27 (67.5%)
3	0 (0%)	0 (0%)	10 (25.0%)
4	0 (0%)	0 (0%)	2 (5.0%)
5	0 (0%)	0 (0%)	1 (2.5%)
Missing	62 (88.6%)	0 (0%)	0 (0%)
**ePAL**			
Mean (SD)	13.9 (6.04)	102 (59.1)	180 (96.8)
Median [Min, Max]	13.0 [5.00, 43.0]	85.0 [55.0, 276]	145 [67.0, 501]
Missing	1 (1.4%)	0 (0%)	1 (2.5%)

**Table 2:** Number of visits across groups.

**Visits**	**All**	**Controls *N***	**PreDM1**	**DM1**
	***N* (%)**	***N* (%)**	***N* (%)**	***N* (%)**
1	123 (41.41)	70 (42.94)	13 (43.33)	40 (38.46)
2	107 (36.03)	59 (36.20)	11 (36.67)	37 (35.58)
3	67 (22.56)	34 (20.86)	6 (20.00)	27 (25.96)

### Plasma Concentrations of Brain Injury Markers Across Groups

**Figure 2:**
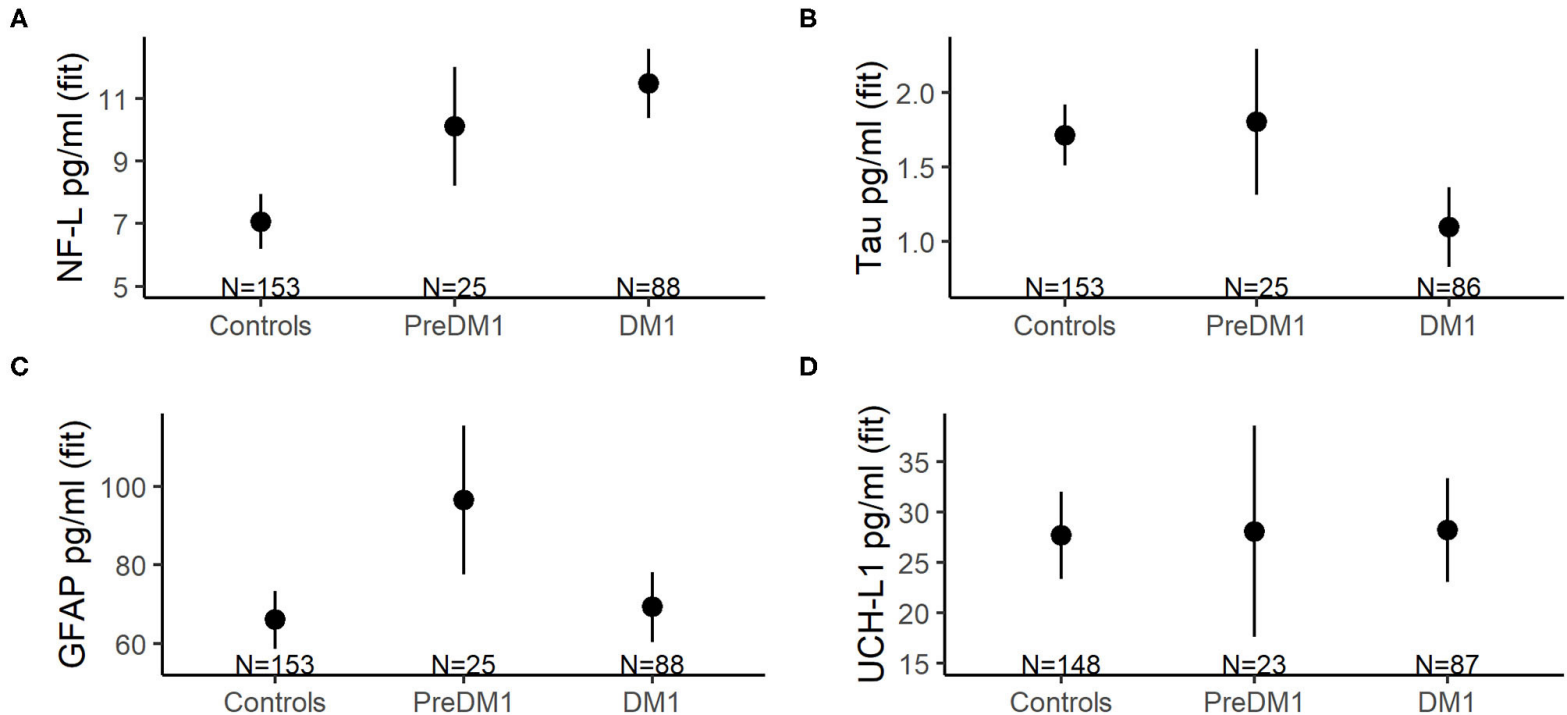
Mean concentrations of NF-L **(A)**, total tau **(B)**, GFAP **(C)**, and UCH-L1 **(D)** across groups (*x*-axes). The group means were derived from mixed linear models that also included main effects for age at evaluation and sex, as well as random intercepts and slopes for age at evaluation, and random effects for participants. The error bars represent 95% confidence limits of the estimated means.

Unadjusted descriptive statistics for NF-L are presented in Table 3. In addition to the random intercepts and slopes, the final model for NF-L included main effects of group, age at evaluation, and sex (Supplementary Table 1). Group significantly predicted NF-L [*F*_(2, 147)_ = 17.99, *P* < 0.0001; Figure 2A], and so did age [*F*_(1, 44)_=20.31, *P* < 0.0001]; however, NF-L was not predicted by sex [*F*_(1, 108)_ = 0.52, *P* = 0.451]. The PreDM1 and manifest DM1 group had significantly higher NF-L relative to controls (DM1 vs. Controls Estimate = 4.43, 95% CI 3.02:5.84, *P* < 0.001; PreDM1 vs. Controls Estimate = 3.04, 95% CI 0.95:5.12, *P* < 0.01).

**Table 3:** Descriptive statistics of blood-based markers of brain injury.

	**Controls**	**PreDM1**	**DM1**	**Overall**
	**(*N =* 163)**	**(*N =* 30)**	**(*N =* 104)**	**(*N =* 297)**
**NFL (pg/ml)**				
Mean (SD)	6.90 (3.16)	10.1 (4.53)	11.9 (5.93)	8.87 (4.96)
Median [Min, Max]	6.24 [1.83, 18.2]	8.96 [3.69, 19.9]	10.9 [3.04, 36.4]	7.96 [1.83, 36.4]
Missing	10 (6.1%)	5 (16.7%)	16 (15.4%)	31 (10.4%)
**Tau (pg/ml)**				
Mean (SD)	1.72 (1.21)	1.89 (1.28)	1.09 (0.853)	1.53 (1.15)
Median [Min, Max]	1.55 [0.130, 11.4]	1.35 [0.229, 5.30]	0.918 [0.146, 5.96]	1.29 [0.130, 11.4]
Missing	10 (6.1%)	5 (16.7%)	18 (17.3%)	33 (11.1%)
**GFAP (pg/ml)**				
Mean (SD)	62.7 (28.8)	104 (73.2)	74.8 (53.9)	70.6 (45.4)
Median [Min, Max]	60.5 [13.6, 208]	96.1 [37.0, 399]	64.0 [21.0, 449]	62.9 [13.6, 449]
Missing	10 (6.1%)	5 (16.7%)	16 (15.4%)	31 (10.4%)
**UCH-L1 (pg/ml)**				
Mean (SD)	27.2 (17.8)	28.6 (9.42)	29.9 (27.7)	28.2 (21.1)
Median [Min, Max]	22.0 [9.48, 142]	26.4 [16.3, 47.7]	24.5 [10.4, 210]	23.5 [9.48, 210]
Missing	15 (9.2%)	7 (23.3%)	17 (16.3%)	39 (13.1%)

Refer to Table 3 for descriptive statistics for total tau. The final total tau models also included main effects of group, age at evaluation and sex (Supplementary Table 2). A significant effect of group was identified [*F*_(2, 109)_ = 7.06, *P* < 0.0001; Figure 2B], but age [*F*_(1, 43)_ = 0.24, *P* = 0.607] and sex [*F*_(1, 103)_ = 1.26, *P* = 0.265] were not significantly associated with total tau. The manifest DM1 group had significantly lower total tau compared with controls (DM1 vs. Controls estimate = −0.62, 95%CI −0.95: −0.28, *P* < 0.001), but the PreDM1 group did not differ from controls (Estimate = 0.09, 95% CI −0.44:0.62, *P* = 0.741). The difference between the DM1 groups was also significant (PreDM1 vs. Controls estimate = −0.46, 95% −0.89: −0.04, *P* = 0.032).

GFAP descriptive statistics are summarized in Table 3. Main effects of group, age at evaluation and sex were included in the final model for GFAP (Supplementary Table 3), and group [*F*_(2, 98)_ = 4.01, *P* = 0.021] and age [*F*_(1, 48)_ = 25.11, *P* < 0.0001] were significantly associated with GFAP, but not sex [F_(1, 75)_ = 2.89, *P* = 0.09]. The group effect was driven by the PreDM1 group (Figure 2C; Controls vs. PreDM1; Estimate = 30.37, 95% CI 10.56–50.19, *P* < 0.01; PreDM1 vs. DM1; estimate = −27.76, 95% CI −54.48:1.04, *P* = 0.042).

Descriptive statistics for UCH-L1 are listed in Table 3. No significant predictors of UCH-L1 were identified (Figure 2D; Supplementary Table 4).

### Impact of EPAL on Brain Injury Markers

**Figure 3:**
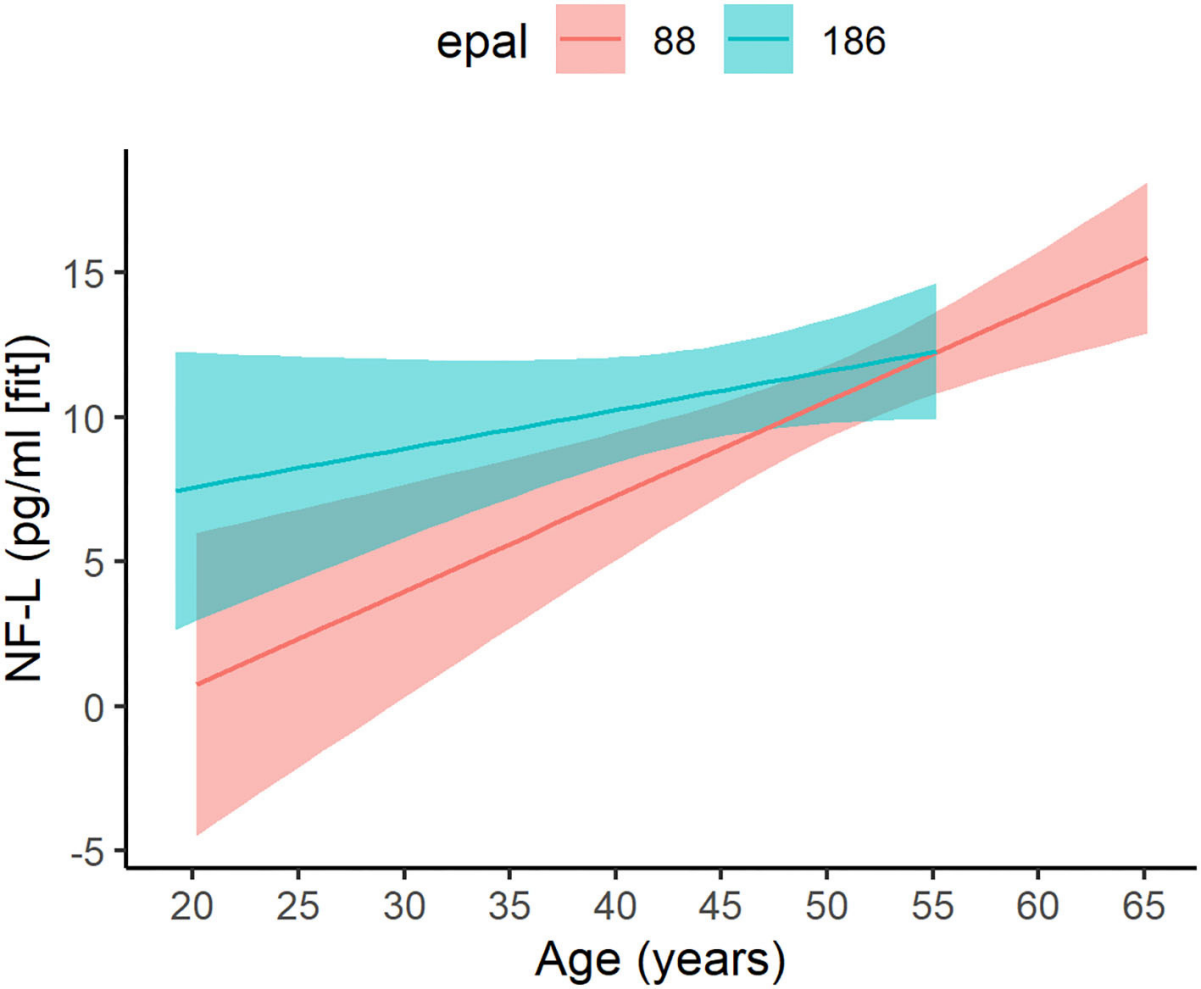
Age-related change in patients with varying ePAL. Age-related change (*x*-axis) of ePAL (*y*-axis) differed for patients with higher repeats (blue) relative to those with lower repeats (pink). To effectively depict the interaction, two representative ePAL repeats were selected based on the median split. The regression lines and confidence limits were derived from a mixed linear model that included random intercepts and slopes for age at evaluation, as well as a random effect for participants. Predictions for the observed age range are included only.

For the ePAL analysis involving individuals with DM1 only, the model with the smallest AICc included an age by ePAL interaction term (Supplementary Table 5). This interaction predicted NF-L levels in DM1 (estimate = −0.0021, 95% CI −0.0042: −0.0001, *P* = 0.045; Figure 3). Higher ePAL was associated with higher NF-L in young participants; however, the rate of age-related change was faster in DM1 patients with a shorter ePAL. Neither tau, GFAP or UCH-L were significantly associated with ePAL (Supplementary Tables 6–8).

### Change in Brain Injury Markers Over Time

Elapsed time was not significantly associated with any of the brain injury markers considered in the analyses (Supplementary Tables 9–12).

### Associations Between White Matter FA and Brain Injury Markers in DM1

**Figure 4:**
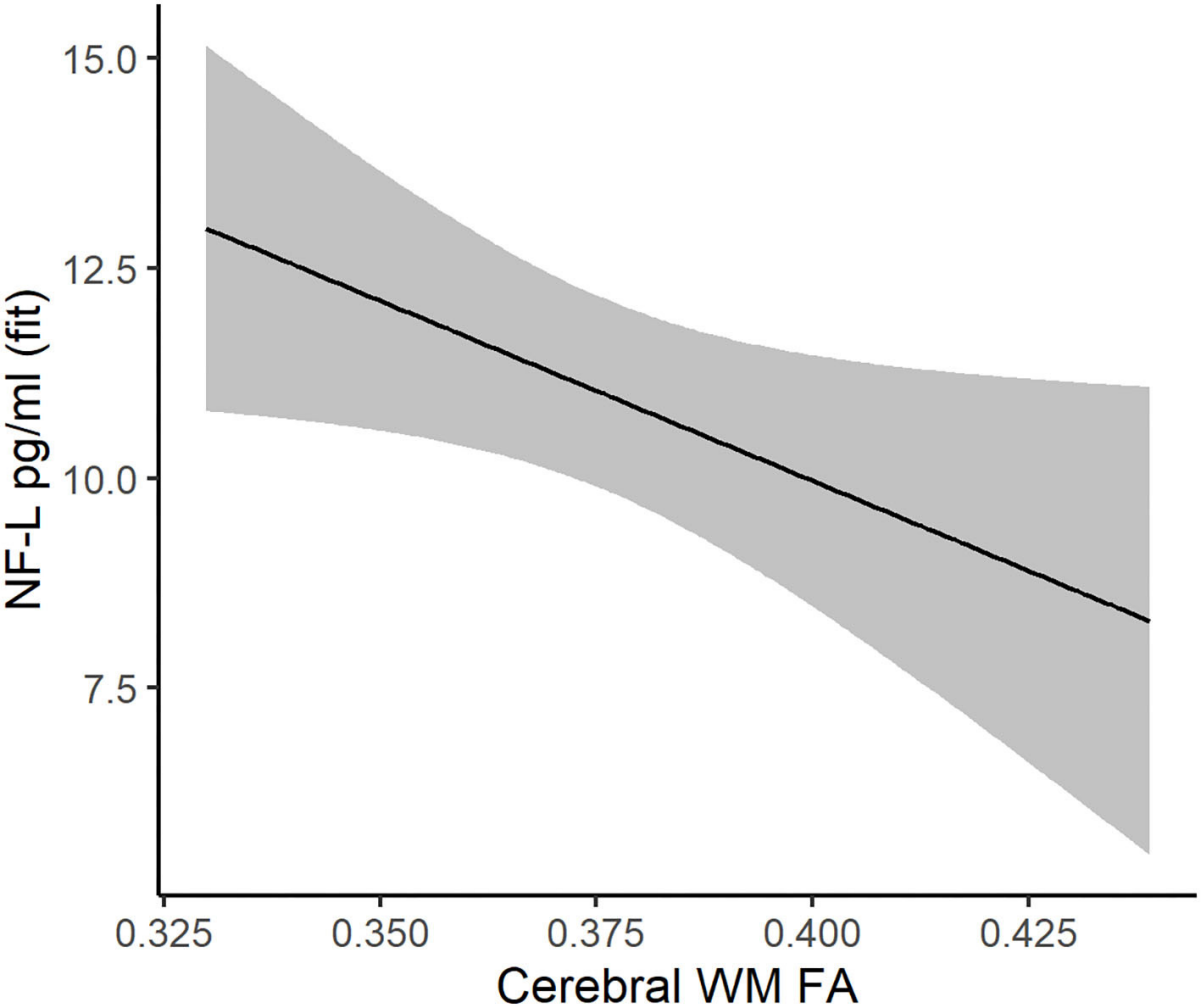
Association between cerebral white matter FA (x-axis) and NF-L (y-axis) in DM1 patients. The regression line and 95% confidence limits were derived from a mixed linear model that also included age at evaluation, sex, and random intercepts and slopes for age at evaluation.

For NF-L, the model with the smallest AICc included main effects of cerebral WM FA, age at assessment, and sex (Supplementary Table 13). Cerebral WM FA was a significant predictor of NF-L, where decreased FA was associated with increased NF-L (estimate = −42.86, 95% CI −82.70: −3.02, *P* = 0.035, Figure 4). Cerebral WM FA was not significantly associated with tau, GFAP, or UCH-L1 (Supplementary Tables 14–16).

## Discussion

While NF-L has been studied extensively in individuals with neurodegenerative illness (12–15, 27); its utility as biomarker of CNS pathology in DM1 had not been determined prior to this study. Plasma NF-L was elevated among individuals with manifest DM1 relative to controls, and those with PreDM1 exhibited intermediate levels compared with controls and manifest DM1 patients. These results imply that plasma NF-L is a sensitive marker of brain pathology and could be useful in predicting disease onset. Neurodegenerative disorders can sometimes be defined by specific protein accumulations, such as α-synuclein in Lewy body disorders or Aβ in Alzheimer's disease; however, neurodegenerative disorders also share fundamental processes (28), including demyelination, dysmyelination, axonal injury, de-arborization and neuronal death. NF-L appears to be a general marker of neuronal pathology that is not specific to a single disorder (12).

Plasma NF-L was associated with disease severity in DM1, as approximated by ePAL and cerebral WM FA. Associations between NF-L and ePAL were dependent on age, where patients with higher ePAL tended to have elevated NF-L at a younger age relative to patients with lower ePAL. However, faster age-related change appeared evident in the latter. Since NF-L is highly expressed in large, myelinated axons (27), it is possible that substantial white matter degeneration among individuals with high ePAL results in plateauing of NF-L levels and subsequent slowing of NF-L release into plasma (14). The analyses on cerebral WM FA confirmed that severity of WM abnormality in DM1 was associated with plasma NF-L. Collectively, it appears that NF-L may be useful in quantifying DM1 disease burden, particularly early on.

Determining markers of disease progression is crucial for the design of clinical trials targeting neurodegeneration (29). The markers evaluated in the present study did not change substantially over the observed period, suggesting that they would not be useful in tracking disease progression among individuals with manifest disease. Our results correspond with work in spinocerebellar ataxia showing that NF-L was stable during the study period, despite clinical progression (14). A study in individuals with HD likewise demonstrated that NF-L was not associated with clinical outcomes in individuals with manifest disease (13). Adult-onset DM1 is characterized by slow clinical disease progression (30), indicating that longer follow-up periods may be required to detect changes in blood-based markers. Our findings of elevated plasma NF-L in PreDM1 individuals suggest that neurodegeneration occurs prior to motor symptoms, supporting the notion that neurons degenerate relatively slowly over the course of the illness. Blood-based markers such as plasma NF-L provide a reliable, non-invasive measure of CNS morbidity; however, further research is required to evaluate if and how blood-based markers track CNS disease progression associated with DM1.

Brain pathology in DM1 is characterized by the presence of neurofibrillary tangles (NFT), distinguishing DM1 as a “tauopathy” (31). Our finding of reduced plasma tau concentrations among DM1 patients contrasted previous research using CSF (16, 17), which may in part be explained by the tissue type used to quantify protein concentrations (i.e., blood vs. CSF). It is also possible that the tau kit did not capture the unique tau expression pattern associated with DM1. The kit is sensitive to the fetal isoform that is overexpressed in DM1 patients (32); however, the detection antibody does not recognize the epitope when tyrosine 18 is phosphorylated. Immunohistochemistry studies have identified that tau proteins are hyperphosphorylated in DM1 (32, 33), and NFTs that have been found in the DM1 brain are the result of aggregation of hyper-phosphorylated tau (34). Further, Peric and colleagues reported evidence of increased phosphorylated tau levels in patients with adult-onset DM1 relative to juvenile-onset DM1 and controls (16). Phosphorylated tyrosine 18 is evident in normal neuronal development (Supplementary Figure 3), which is notable with regard to overexpression of the fetal tau isoform (N0) in DM1 (32). It is possible that the N0 tau isoform is more prone to be phosphorylated at tyrosine 18 than the N1 and N2 tau isoforms. Considering the available evidence, we postulate that tyrosine 18 is abnormally phosphorylated in DM1, in which case our assay would be expected to have the lowest sensitivity in patients with the highest levels of tau pathology by way of increased phosphorylation.

GFAP is an intermediate filament protein expressed in astrocytes (35). Expression of GFAP is known to increase with age, which was also evident in the present study. However, we did not expect GFAP levels to be elevated in PreDM1 only. During neurodegenerative disease, astrocytes become reactive and gain abnormal roles that include enhanced GFAP expression (18, 36). Histopathological findings in DM1 brain tissue also shows evidence of gliosis (34). Our findings may reflect temporally dynamic changes in GFAP over the course of DM1 progression. Further research is required to understand the role of GFAP in DM1 brain pathology.

Some limitations should be considered. First, DM1 is a rare disorder, and our sample size was relatively limited, particularly regarding PreDM1 individuals. Sample limitations may have affected our ability to detect changes in marker concentrations. Second, manifest DM1 patients in the present sample were also relatively mildly affected, with most manifest patients having a MIRS of two. Our sample composition may have further complicated our ability to detect markers of disease progression. Third, NF-L concentrations are influenced by factors beyond neurodegeneration, including cardiovascular risk factors and body mass index (BMI) (12). However, the groups did not differ significantly in BMI (*P* = 0.807). Moreover, the demonstrated association between NF-L and cerebral WM FA underscores the role of CNS pathology in NF-L levels in DM1. It should also be noted that studies in HD have demonstrated that plasma levels of NF-L are highly correlated to CSF-derived NF-L concentrations (15). Finally, the sample included more women than men across groups. We evaluated the impact of sex in our models to account for a possible bias related to sex. Nonetheless, replication of our findings is warranted.

In conclusion, NF-L was sensitive to disease severity of DM1, and may potentially be useful in predicting disease onset; however, the utility of NF-L as an endpoint in clinical trials remains to be elucidated. The observed reduction in total tau in DM1 patients may be the result of tyrosine 18 phosphorylation in DM1, which cannot be detected with the kit that was used. The mechanisms of CNS morbidity in DM1 are complex and largely unexplored (37). Expanding the scope and depth of biomarkers research offers opportunities for gaining insight into the neuropathological processes of CNS morbidity associated with DM1.

## Data Availability Statement

De-identified data may be shared upon reasonable request to the corresponding author.

## Ethics Statement

The studies involving human participants were reviewed and approved by The University of Iowa Institutional Review Board. The patients/participants provided their written informed consent to participate in this study.

## Author Contributions

PN and LG: contributed the conception and design of the study. EvdP, JL, TK, VM, DM, SC, AG, and MH: contributed to acquisition and analysis of the data. EvdP, JL, TK, VM, DM, SC, AG, MH, LG, and PN: contributed to drafting significant portion of the manuscript and/or figures. All authors contributed to the article and approved the submitted version.

## Funding

This work was supported by the National Institute of Neurological Disorders and Stroke (R01 NS094387, PN). The MRI instrument was funded by 1S10OD025025-01.

## Conflict of Interest

EvdP, TK, VM, SC, AG, MH, LG, and PN all report no disclosures. JL is a paid advisory board member for F. Hoffmann-La Roche Ltd and UniQue, and he is a paid consultant for Triplet, PTC, and Remix. DM has been a scientific consultant and/or received an honoraria or stock options from Biogen Idec, AMO Pharma, Charles River, Vertex Pharmaceuticals, Triplet Therapeutics, LoQus23, BridgeBio, and Small Molecule RNA. DM is on the Scientific Advisory Board of the Myotonic Dystrophy Foundation, is a scientific advisor to the Myotonic Dystrophy Support Group and is a vice president of Muscular Dystrophy UK.

## Publisher's Note

All claims expressed in this article are solely those of the authors and do not necessarily represent those of their affiliated organizations, or those of the publisher, the editors and the reviewers. Any product that may be evaluated in this article, or claim that may be made by its manufacturer, is not guaranteed or endorsed by the publisher.
