# Supplementary material for: Blood-Based Markers of Neuronal Injury in Adult-Onset Myotonic Dystrophy Type 1
Source: Front Neurol. 2022 Jan 20;12:791065. doi: 10.3389/fneur.2021.791065 (PMC8810511; doi:10.3389/fneur.2021.791065)
Supplement: Supplementary file 1 [file Data_Sheet_1.docx]

# Supplementary materials

## eFigure 1 Density plot showing overlapping distributions of scanner version and vendor (colored lines)

Scanner software version or vendor was not significantly associated with cerebral WM FA (*F*_(1, 4)_=0.598, *P*=.664)


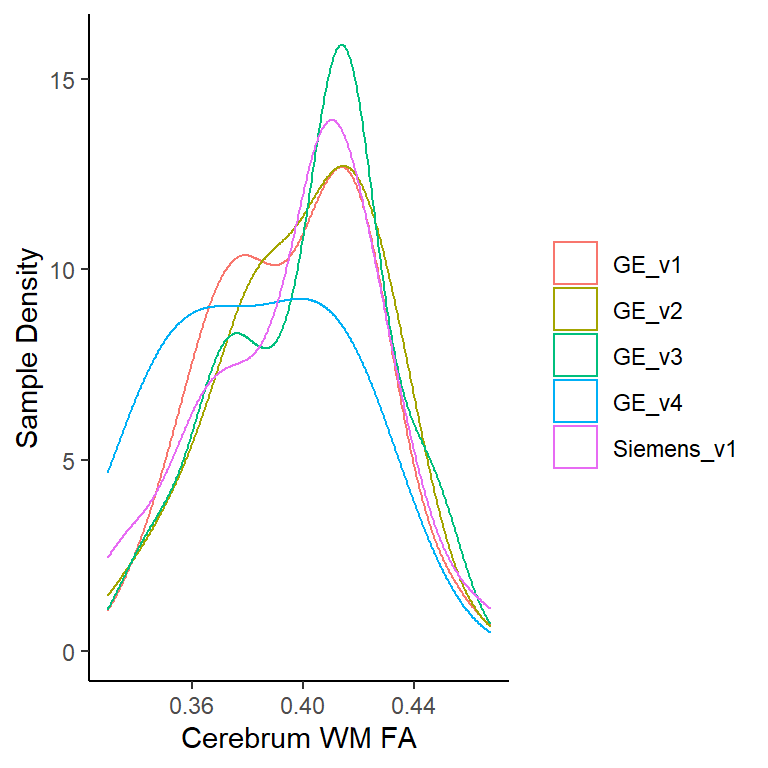


## eFigure 2 QQplots for outcome measures across groups

Q-QPlots were used to inspect the distribution of the outcome measures across groups. While there was some deviation from the normal distribution, it should be noted that our statistical models can provide reliable results in the context of such degree of deviation from the normal distribution.^1^
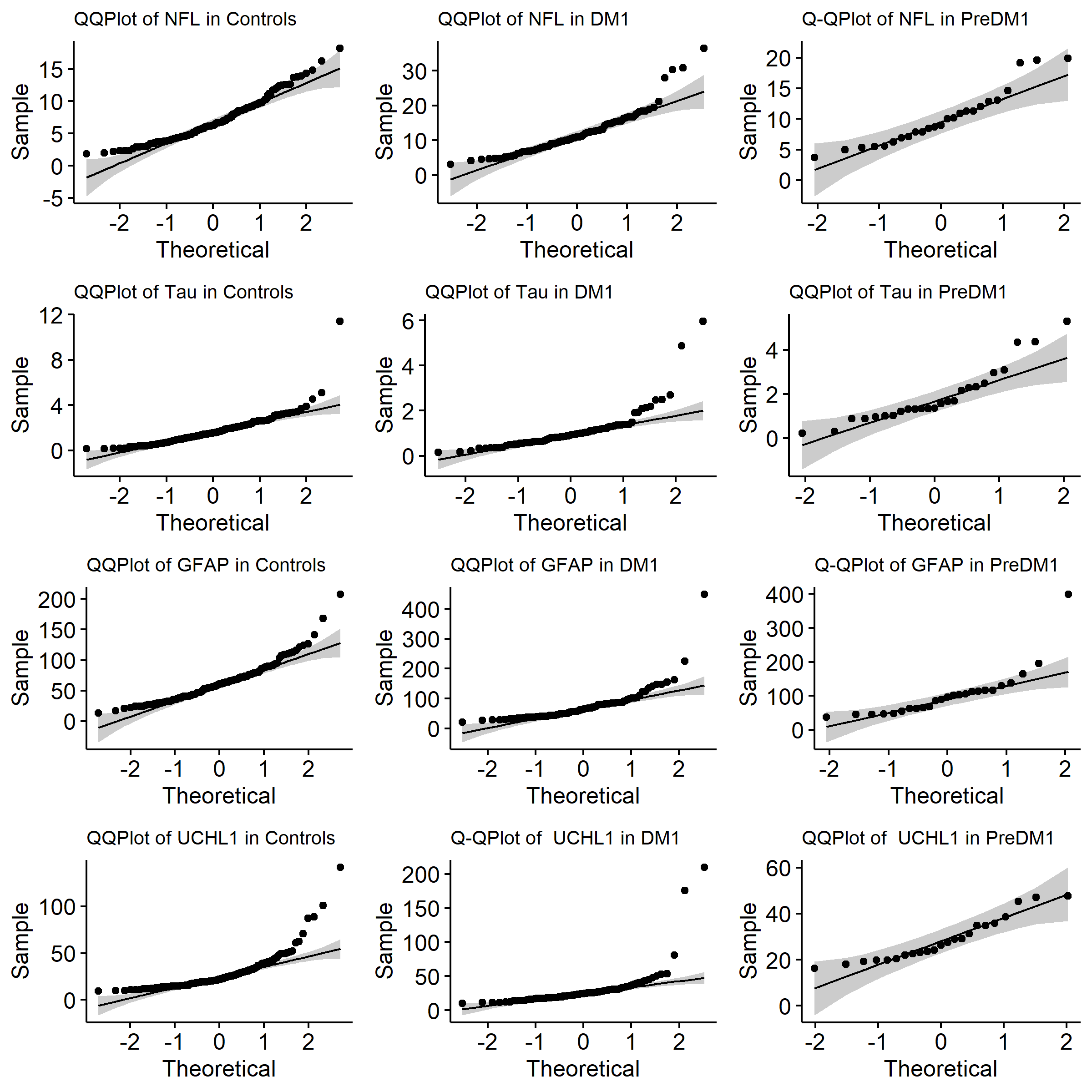


## eFigure 3 Phosphorylated tyrosine 18 expression in early brain development

De-identified frozen brain tissue from the frontoparietal cortex of two 19-week human fetuses with no known genetic disease, malformation or gross neuropathologic abnormalities were obtained from the NIH NeuroBioBank. In compliance with Iowa law, no tissue from elective terminations were used. Tissue was homogenized and fractionated by solubility as described in previously.^2^ We used the first step of the protocol to obtain the soluble fraction, the supernatant from first round of the 1% Triton X-100 steps for the detergent-soluble fraction, and the last step of the protocol for the insoluble fraction. These were then loaded onto SDS-PAGE gels, transferred and separate gels were probed with either anti-pTyr18 (9G3) or total tau (HT7) antibodies.


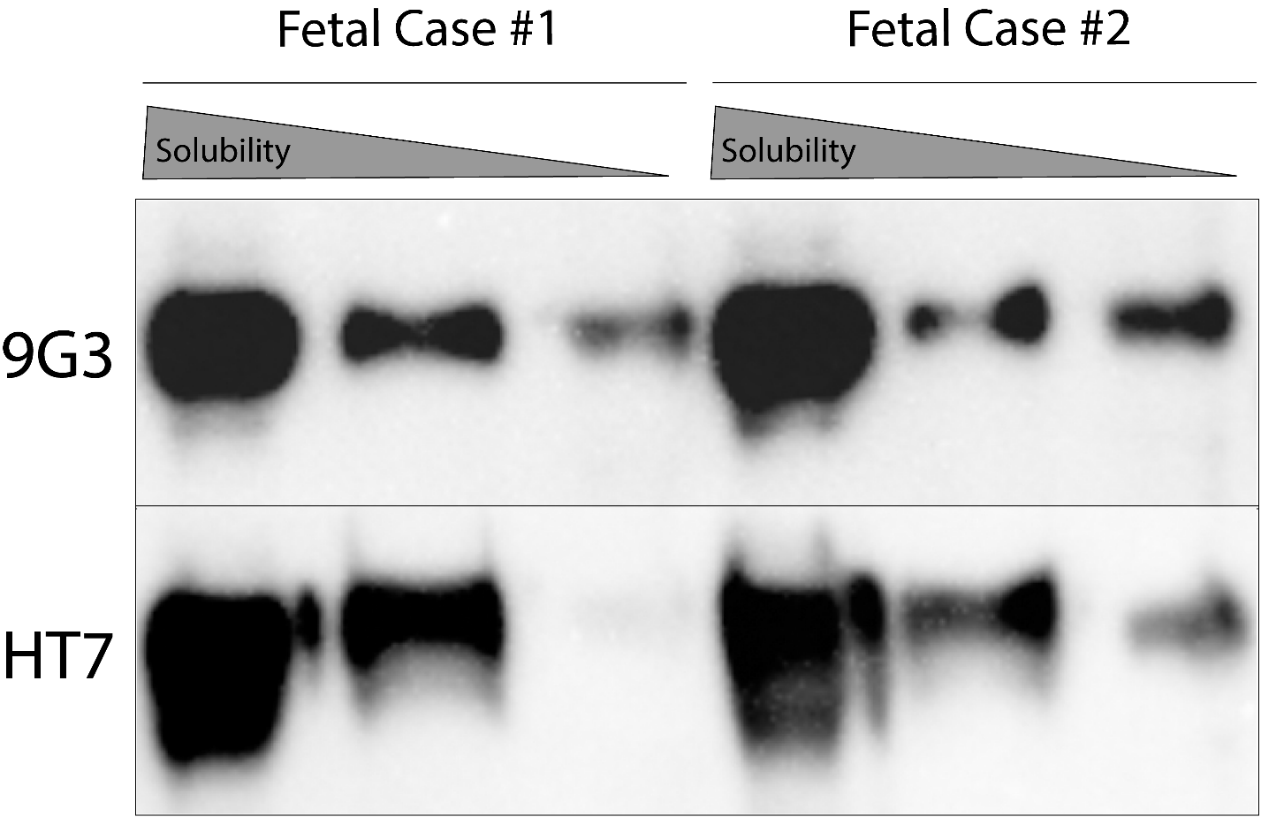


# Supplementary Tables

## eTable 1 AIC table for NF-L

| *Modnames* | *K* | *AICc* | *Delta_AICc* | *ModelLik* | *AICcWt* | *LL* |
| --- | --- | --- | --- | --- | --- | --- |
| nfl1 | 7 | 1469.72 | 29.13 | 0.00 | 0.00 | -727.64 |
| **nfl2** | **9** | **1440.59** | **0.00** | **1.00** | **0.83** | **-710.94** |
| nfl3 | 11 | 1443.81 | 3.22 | 0.20 | 0.17 | -710.39 |

nfl1: Main effect of age at evaluation, and sex; nfl2: main effects of group, age at evaluation and sex; nfl3: group*age interaction and main effect of sex. All models included random intercepts and random slopes for age, as well as random effects of participant. The model that was selected (delta AIC=0) is highlighted in bold.

## eTable 2 AIC table for total tau

| *Modnames* | *K* | *AICc* | *Delta_AICc* | *ModelLik* | *AICcWt* | *LL* |
| --- | --- | --- | --- | --- | --- | --- |
| tau1 | 7 | 820.19 | 10.14 | 0.01 | 0.00 | -402.88 |
| **tau2** | **9** | **810.06** | **0.00** | **1.00** | **0.54** | **-395.67** |
| tau3 | 11 | 810.37 | 0.31 | 0.86 | 0.46 | -393.66 |

tau1: Main effect of age at evaluation, and sex; tau2: main effects of group, age at evaluation, and sex; tau3: group*age interaction and main effect of sex. All models included random intercepts and random slopes for age, as well as random effects of participant. The model that was selected (delta AIC=0) is highlighted in bold.

## eTable 3 AIC table for GFAP

| *Modnames* | *K* | *AICc* | *Delta_AICc* | *ModelLik* | *AICcWt* | *LL* |
| --- | --- | --- | --- | --- | --- | --- |
| gfap1 | 7 | 2669.92 | 4.79 | 0.09 | 0.05 | -1327.74 |
| **gfap2** | **9** | **2665.12** | **0.00** | **1.00** | **0.50** | **-1323.21** |
| gfap3 | 11 | 2665.32 | 0.20 | 0.91 | 0.45 | -1321.14 |

gfap1: Main effect of age at evaluation, and sex; gfap2: main effects of group, age at evaluation, and gender; gfap3: group*age interaction and main effect of sex. All models included random intercepts and random slopes for age, as well as random effects of participant. The model that was selected (delta AIC=0) is highlighted in bold.

## eTable 4 AIC table for UCH-L1

| Modnames | K | AICc | Delta_AICc | ModelLik | AICcWt | LL |
| --- | --- | --- | --- | --- | --- | --- |
| **uchl1** | **7** | **2230.08** | **0.00** | **1.00** | **0.81** | **-1107.81** |
| uchl2 | 9 | 2234.32 | 4.25 | 0.12 | 0.10 | -1107.80 |
| uchl3 | 9 | 2234.32 | 4.25 | 0.12 | 0.10 | -1107.80 |

uchl1: Main effect of age at evaluation, and sex; uchl2: main effects of group, age at evaluation and sex; uchl3: group*age interaction and main effect of sex. All models included random intercepts and random slopes for age, as well as random effects of participant. The model that was selected (delta AIC=0) is highlighted in bold.

## eTable 5 Model selection for NF-L and ePAL

| *Modnames* | *K* | *AICc* | *Delta_AICc* | *ModelLik* | *AICcWt* | *LL* |
| --- | --- | --- | --- | --- | --- | --- |
| **nfl.age.epal1** | **8** | **653.75** | **0.00** | **1.00** | **0.56** | **-318.16** |
| nfl.age.epal2 | 7 | 655.64 | 1.90 | 0.39 | 0.22 | -320.27 |
| nfl.age.epal3 | 6 | 676.79 | 23.05 | 0.00 | 0.00 | -332.00 |
| wm.uchl1.fa4 | 10 | 656.33 | 2.59 | 0.27 | 0.15 | -317.05 |
| wm.uchl1.fa5 | 8 | 657.88 | 4.13 | 0.13 | 0.07 | -320.23 |
| nfl.age.epal6 | 7 | 678.29 | 24.55 | 0.00 | 0.00 | -331.61 |

nfl.age.epal1 included an age at evaluation*ePAL interaction; nfl.age.epal2 included main effects for age at evaluation, and ePAL; nfl.age.epal3 included a main effect of age at evaluation; nfl.age.epal4 included a polynomial term for age at evaluation*ePAL interaction; nfl.age.epal5 included a polynomial term for age at evaluation, and a main effect of ePAL; nfl.age.epal6 included a polynomial term for age at evaluation; All models included random intercepts and random slopes for age at evaluation, as well as random effects of participant. The model that was selected (delta AIC=0) is highlighted in bold.

## eTable 6 Model selection for total tau and ePAL

| *Modnames* | *K* | *AICc* | *Delta_AICc* | *ModelLik* | *AICcWt* | *LL* |
| --- | --- | --- | --- | --- | --- | --- |
| tau.age.epal1 | 8 | 296.53 | 2.32 | 0.31 | 0.16 | -139.54 |
| **tau.age.epal2** | **7** | **294.21** | **0.00** | **1.00** | **0.51** | **-139.54** |
| tau.age.epal3 | 6 | 298.26 | 4.05 | 0.13 | 0.07 | -142.73 |
| wm.uchl1.fa4 | 10 | 300.43 | 6.22 | 0.04 | 0.02 | -139.08 |
| wm.uchl1.fa5 | 8 | 295.97 | 1.76 | 0.42 | 0.21 | -139.26 |
| tau.age.epal6 | 7 | 299.80 | 5.59 | 0.06 | 0.03 | -142.36 |

tau.age.epal1 included an age at evaluation*ePAL interaction; tau.age.epal2 included main effects for age at evaluation, and ePAL; tau.age.epal3 included a main effect of age at evaluation; tau.age.epal4 included a polynomial term of age at evaluation*ePAL interaction; tau.age.epal5 included a polynomial term of age at evaluation, and a main effect of ePAL; tau.age.epal6 included a polynomial term of age at evaluation. All models included random intercepts and random slopes for age, as well as random effects of participant. The model that was selected (AIC=0) is highlighted in bold. None of the predictors were significantly associated with total tau (age estimate=-0.01, 95% CI -0.04-0.01, *P*=.370; ePAL estimate= -0.0020; 95% CI -0.0049:0.0010, *P=*.189).

## eTable 7 Model selection for GFAP and ePAL

| *Modnames* | *K* | *AICc* | *Delta_AICc* | *ModelLik* | *AICcWt* | *LL* |
| --- | --- | --- | --- | --- | --- | --- |
| **gfap.age.epal1** | **8** | **1178.91** | **0.00** | **1.00** | **0.44** | **-580.74** |
| gfap.age.epal2 | 7 | 1179.13 | 0.22 | 0.89 | 0.40 | -582.02 |
| gfap.age.epal3 | 6 | 1207.72 | 28.81 | 0.00 | 0.00 | -597.46 |
| wm.uchl1.fa4 | 10 | 1183.72 | 4.81 | 0.09 | 0.04 | -580.75 |
| wm.uchl1.fa5 | 8 | 1181.55 | 2.64 | 0.27 | 0.12 | -582.06 |
| gfap.age.epal6 | 7 | 1210.00 | 31.09 | 0.00 | 0.00 | -597.46 |

gfap.age.epal1 included an age at evaluation*ePAL interaction; gfap.age.epal2 included main effects for age at evaluation, and ePAL; gfap.age.epal3 included a main effect of age at evaluation; gfap.age.epal4 included a polynomial term for age at evaluation*ePAL interaction; gfap.age.epal5 included a polynomial term for age at evaluation, and a main effect of ePAL; gfap.age.epal6 included a polynomial term for age at evaluation. All models included random intercepts and random slopes for age, as well as random effects of participant. The model that was selected (delta AIC=0) is highlighted in bold. The age*ePAL interaction was not significantly associated with GFAP (Interaction estimate: -0.02, 95% CI -0.0382:0.0042, *P=*.117).

## eTable 8 Model selection for UCH-L1 and ePAL

| Modnames | K | AICc | Delta_AICc | ModelLik | AICcWt | LL |
| --- | --- | --- | --- | --- | --- | --- |
| uchl1.age.epal1 | 8 | 926.31 | 2.23 | 0.33 | 0.18 | -454.42 |
| **uchl1.age.epal2** | **7** | **924.08** | **0.00** | **1.00** | **0.56** | **-454.48** |
| uchl1.age.epal3 | 6 | 951.91 | 27.83 | 0.00 | 0.00 | -469.55 |
| wm.uchl1.fa4 | 10 | 929.53 | 5.45 | 0.07 | 0.04 | -453.62 |
| wm.uchl1.fa5 | 8 | 925.92 | 1.84 | 0.40 | 0.22 | -454.22 |
| uchl1.age.epal6 | 7 | 953.33 | 29.25 | 0.00 | 0.00 | -469.12 |

uchl1.age.epal1 included an age at evaluation*ePAL interaction; uchl1.age.epal2 included main effects for age at evaluation, and ePAL; uchl1.age.epal3 included a main effect of age at evaluation; uchl1.age.epal4 included a polynomial term for age at evaluation*ePAL interaction; uchl1.age.epal5 included a polynomial term for age at evaluation, and a main effect of ePAL; uchl1.age.epal6 included a polynomial term for age at evaluation. All models included random intercepts and random slopes for age, as well as random effects of participant. The model that was selected (delta AIC=0) is highlighted in bold. None of the predictors were significantly associated with UCH-L1 (Age Estimate=0.49, 95% CI -0.108:-1.08, *P*=.108; ePAL Estimate=0.02, 95% CI -0.03:0.07, *P*=.400).

## eTable 9 Model selection for elapsed time and NF-L.

| *Modnames* | *K* | *AICc* | *Delta_AICc* | *ModelLik* | *AICcWt* | *LL* |
| --- | --- | --- | --- | --- | --- | --- |
| nfl.c0 | 7 | 684.97 | 3.71 | 0.16 | 0.07 | -334.95 |
| **nfl.c1** | **8** | **681.26** | **0.00** | **1.00** | **0.48** | **-331.94** |
| nfl.c2 | 9 | 682.19 | 0.93 | 0.63 | 0.30 | -331.22 |
| nfl.c3 | 10 | 684.08 | 2.81 | 0.24 | 0.12 | -330.96 |
| nfl.c4 | 11 | 686.49 | 5.23 | 0.07 | 0.03 | -330.94 |

nfl.c0 included main effects for years on study, and sex; nfl.c1 included main effects for years on study, and age at baseline; nfl.c2 included group (PreDM1 vs. DM1), years on study, age at baseline, and sex; nfl.c3 included a group*years on study interaction, and main effects of age at baseline, and sex; and nfl.c4 included group*years on study, and group*age at baseline interactions, and a main effect of sex. All models included random intercepts and random slopes for elapsed time, as well as random effects of participant. The model that was selected (delta AIC=0) is highlighted in bold. Elapsed time was not significantly associated with NF-L (Estimate=0.05, 95% CI -0.64:0.73, *P*=.895).

## eTable 10 Model selection for elapsed time and total tau.

| *Modnames* | *K* | *AICc* | *Delta_AICc* | *ModelLik* | *AICcWt* | *LL* |
| --- | --- | --- | --- | --- | --- | --- |
| tau.c0 | 7 | 312.10 | 6.98 | 0.03 | 0.02 | -148.50 |
| tau.c1 | 8 | 312.13 | 7.01 | 0.03 | 0.02 | -147.36 |
| tau.c2 | 9 | 307.67 | 2.55 | 0.28 | 0.19 | -143.94 |
| tau.c3 | 10 | 309.35 | 4.23 | 0.12 | 0.08 | -143.58 |
| **tau.c4** | **11** | **305.12** | **0.00** | **1.00** | **0.68** | **-140.23** |

tau.c0 included main effects for years on study, and sex; tau.c1 included main effects for years on study, and age at baseline; tau.c2 included group (PreDM1 vs. DM1), years on study, age at baseline, and sex; tau.c3 included a group*years on study interaction, and main effects of age at baseline, and sex; and tau.c4 included group*years on study, and group*age at baseline interactions, and a main effect of sex. All models included random intercepts and random slopes for elapsed time, as well as random effects of participant. The model that was selected (delta AIC=0) is highlighted in bold. The group*years on study was not significantly associated with NF-L (Estimate=-0.16, 95% CI -0.54:0.22, *P*=.395).

## eTable 11 Model selection for elapsed time and GFAP.

| *Modnames* | *K* | *AICc* | *Delta_AICc* | *ModelLik* | *AICcWt* | *LL* |
| --- | --- | --- | --- | --- | --- | --- |
| gfap.c0 | 7 | 1216.89 | 4.34 | 0.11 | 0.05 | -600.91 |
| **gfap.c1** | **8** | **1212.55** | **0.00** | **1.00** | **0.40** | **-597.58** |
| gfap.c2 | 9 | 1212.88 | 0.33 | 0.85 | 0.34 | -596.57 |
| gfap.c3 | 10 | 1214.40 | 1.85 | 0.40 | 0.16 | -596.12 |
| gfap.c4 | 11 | 1216.50 | 3.95 | 0.14 | 0.06 | -595.95 |

gfap.c0 included main effects for years on study, and sex; gfap.c1 included main effects for years on study, and age at baseline; gfap.c2 included group (PreDM1 vs. DM1), years on study, age at baseline, and sex; gfap.c3 included a group*years on study interaction, and main effects of age at baseline, and sex; and gfap.c4 included group*years on study, and group*age at baseline interactions, and a main effect of sex. All models included random intercepts and random slopes for elapsed time, as well as random effects of participant. The model that was selected (delta AIC=0) is highlighted in bold. Elapsed time was not significantly associated with GFAP (Estimate=-5.36, 95% CI -12.45:1.74, *P*=.139).

## eTable 12 Model selection for elapsed time and UCH-L1.

| Modnames | K | AICc | Delta_AICc | ModelLik | AICcWt | LL |
| --- | --- | --- | --- | --- | --- | --- |
| **uchl1.c0** | **7** | **963.87** | **0.00** | **1.00** | **0.48** | **-474.39** |
| uchl1.c1 | 8 | 965.76 | 1.89 | 0.39 | 0.19 | -474.17 |
| uchl1.c2 | 9 | 965.56 | 1.69 | 0.43 | 0.21 | -472.88 |
| uchl1.c3 | 10 | 967.68 | 3.81 | 0.15 | 0.07 | -472.73 |
| uchl1.c4 | 11 | 968.55 | 4.69 | 0.10 | 0.05 | -471.93 |

uchl1.c0 included main effects for years on study, and sex; uchl1.c1 included main effects for years on study, and age at baseline; uchl1.c2 included group (PreDM1 vs. DM1), years on study, age at baseline, and sex; uchl1.c3 included a group*years on study interaction, and main effects of age at baseline, and sex; and uchl1.c4 included group*years on study, and group*age at baseline interactions, and a main effect of sex. All models included random intercepts and random slopes for elapsed time, as well as random effects of participant. The model that was selected (delta AIC=0) is highlighted in bold. Elapsed time was significantly associated with UCH-L1 (Estimate=-1.53, 95% CI -3.73:0.67, *P*=0.173).

## eTable 13 Model selection for cerebral WM FA and NF-L

| *Modnames* | *K* | *AICc* | *Delta_AICc* | *ModelLik* | *AICcWt* | *LL* |
| --- | --- | --- | --- | --- | --- | --- |
| wm.fa0 | 6 | 676.79 | 81.49 | 0.00 | 0.00 | -332.00 |
| **wm.fa1** | **8** | **595.31** | **0.00** | **1.00** | **0.70** | **-288.88** |
| wm.fa2 | 9 | 597.71 | 2.41 | 0.30 | 0.21 | -288.88 |
| wm.fa3 | 10 | 600.13 | 4.83 | 0.09 | 0.06 | -288.86 |
| wm.fa4 | 11 | 601.92 | 6.61 | 0.04 | 0.03 | -288.49 |

wm.fa0 included a main effect of age at evaluation; wm.fa1 included main effects of cerebral WM FA, age at evaluation, and sex; wm.fa2 included main effects of cerebral WM FA, group (PreDM1 vs. DM1), age, and sex; wm.fa3 included a cerebral WM FA*age at evaluation interaction, and a main effect of sex; wm.fa4 included group*cerebral WM FA and group*age at evaluation interactions, and a main effect of sex. All models included random intercepts and random slopes for age, as well as random effects of participant. The model that was selected (delta AIC=0) is highlighted in bold.

## eTable 14 Model selection for cerebral WM FA and total tau

| *Modnames* | *K* | *AICc* | *Delta_AICc* | *ModelLik* | *AICcWt* | *LL* |
| --- | --- | --- | --- | --- | --- | --- |
| wm.tau.fa0 | 6 | 298.26 | 47.17 | 0.00 | 0.00 | -142.73 |
| wm.tau.fa1 | 8 | 257.05 | 5.96 | 0.05 | 0.03 | -119.74 |
| **wm.tau.fa2** | **9** | **251.09** | **0.00** | **1.00** | **0.66** | **-115.55** |
| wm.tau.fa3 | 10 | 253.10 | 2.01 | 0.37 | 0.24 | -115.32 |
| wm.tau.fa4 | 11 | 255.83 | 4.74 | 0.09 | 0.06 | -115.42 |

wm.tau.fa0 included a main effect of age at evaluation; wm.tau.fa1 included main effects of cerebral WM FA, age at evaluation, and sex; wm.tau.fa2 included main effects of cerebral WM FA, group (PreDM1 vs. DM1), age, and sex; wm.tau.fa3 included a cerebral WM FA*age at evaluation interaction, and a main effect of sex; wm.tau.fa4 included group*cerebral WM FA and group*age at evaluation interactions, and a main effect of sex. All models included random intercepts and random slopes for age, as well as random effects of participant. The model that was selected (delta AIC=0) is highlighted in bold. Cerebral WM FA was not significantly associated with total tau (Estimate=5.66, 95% CI -2.27:13.59, *P*=.162).

## eTable 15 Model selection for cerebral WM FA and GFAP

| *Modnames* | *K* | *AICc* | *Delta_AICc* | *ModelLik* | *AICcWt* | *LL* |
| --- | --- | --- | --- | --- | --- | --- |
| wm.gfap.fa0 | 6 | 1207.72 | 104.25 | 0.00 | 0.00 | -597.46 |
| wm.gfap.fa1 | 8 | 1103.97 | 0.50 | 0.78 | 0.35 | -543.21 |
| **wm.gfap.fa2** | **9** | **1103.47** | **0.00** | **1.00** | **0.46** | **-541.76** |
| wm.gfap.fa3 | 10 | 1105.93 | 2.46 | 0.29 | 0.13 | -541.76 |
| wm.gfap.fa4 | 11 | 1107.62 | 4.15 | 0.13 | 0.06 | -541.34 |

wm.gfap.fa0 included a main effect of age at evaluation; wm.gfap.fa1 included main effects of cerebral WM FA, age at evaluation, and sex; wm.gfap.fa2 included main effects of cerebral WM FA, group (PreDM1 vs. DM1), age, and sex; wm.gfap.fa3 included a cerebral WM FA*age at evaluation interaction, and a main effect of sex; wm.gfap.fa4 included group*cerebral WM FA and group*age at evaluation interactions, and a main effect of sex. All models included random intercepts and random slopes for age, as well as random effects of participant. The model that was selected (delta AIC=0) is highlighted in bold. Cerebral WM FA was not significantly associated with GFAP (Estimate=24.51, 95% CI -461.64:510.66, *P*=.921).

## eTable 16 Model selection for cerebral WM FA and UCH-L1

| Modnames | K | AICc | Delta_AICc | ModelLik | AICcWt | LL |
| --- | --- | --- | --- | --- | --- | --- |
| wm.uchl1.fa0 | 6 | 951.91 | 104.96 | 0.00 | 0.00 | -469.55 |
| **wm.uchl1.fa1** | **8** | **846.95** | **0.00** | **1.00** | **0.45** | **-414.67** |
| wm.uchl1.fa2 | 9 | 849.12 | 2.17 | 0.34 | 0.15 | -414.55 |
| wm.uchl1.fa3 | 10 | 851.38 | 4.43 | 0.11 | 0.05 | -414.44 |
| wm.uchl1.fa4 | 11 | 847.45 | 0.50 | 0.78 | 0.35 | -411.2 |

wm.uchl1.fa0 included a main effect of age at evaluation; wm.uchl1.fa1 included main effects of cerebral WM FA, age at evaluation, and sex; wm.uchl1.fa2 included main effects of cerebral WM FA, group (PreDM1 vs. DM1), age, and sex; wm.uchl1.fa3 included a cerebral WM FA*age at evaluation interaction, and a main effect of sex; wm.uchl1.fa4 included group*cerebral WM FA and group*age at evaluation interactions, and a main effect of sex. All models included random intercepts and random slopes for age, as well as random effects of participant. The model that was selected (delta AIC=0) is highlighted in bold. WM FA was not significantly associated with UCH-L1 (Estimate=63.52, 95% CI -98.77:225.81, *P*=.443).

# eTable 17 Descriptive statistics across groups and visits

|  | **Controls** | | | **PreDM1** | | | **DM1** | | |
| --- | --- | --- | --- | --- | --- | --- | --- | --- | --- |
|  | **1 (N=70)** | **2 (N=59)** | **3 (N=34)** | **1 (N=13)** | **2 (N=11)** | **3 (N=6)** | **1 (N=40)** | **2 (N=37)** | **3 (N=27)** |
| **Age (years)** |  |  |  |  |  |  |  |  |  |
| Mean (SD) | 43.6 (12.8) | 44.4 (12.6) | 46.2 (13.7) | 47.4 (16.3) | 47.9 (17.5) | 44.9 (17.1) | 46.0 (9.42) | 47.6 (8.95) | 48.3 (10.3) |
| Median  [Min, Max] | 43.7  [18.3, 63.4] | 44.8  [19.6, 64.4] | 48.9  [21.4, 66.0] | 53.3  [19.2, 64.0] | 54.7  [20.5, 65.5] | 50.0  [23.7, 61.8] | 46.1  [30.3, 62.2] | 47.8 [31.4, 63.3] | 49.3 [21.5, 65.1] |
| **Sex** |  |  |  |  |  |  |  |  |  |
| Female | 45 64.3%) | 36 (61.0%) | 20 (58.8%) | 7 (53.8%) | 5 (45.5%) | 3 (50.0%) | 28 (70.0%) | 27 (73.0%) | 18 (66.7%) |
| Male | 25 35.7%) | 23 (39.0%) | 14 (41.2%) | 6 (46.2%) | 6 (54.5%) | 3 (50.0%) | 12 (30.0%) | 10 (27.0%) | 9 (33.3%) |
| **Disease Duration** |  |  |  |  |  |  |  |  |  |
| Mean (SD) | - | - | - | - | - | - | 12.9 (7.29) | 14.0 (7.88) | 13.0 (7.83) |
| Median [Min, Max] | - | - | - | - | - | - | 12.8  [2.42, 28.9] | 13.9  [0.69, 30.4] | 13.8  [0.09, 27.1] |
| Missing | - | - | - | - | - | - | 3 (7.5%) | 2 (5.4%) | 0 (0%) |
| **MIRS** |  |  |  |  |  |  |  |  |  |
| 1 | 7 (10.0%) | 5 (8.5%) | 5 (14.7%) | 13 (100%) | 10 (90.9%) | 6 (100%) | 0 (0%) | 3 (8.1%) | 1 (3.7%) |
| 2 | 1 (1.4%) | 0 (0%) | 0 (0%) | 0 (0%) | 0 (0%) | 0 (0%) | 27 (67.5%) | 20 (54.1%) | 14 (51.9%) |
| 3 | 0 (0%) | 0 (0%) | 0 (0%) | 0 (0%) | 0 (0%) | 0 (0%) | 10 (25.0%) | 9 (24.3%) | 7 (25.9%) |
| 4 | 0 (0%) | 0 (0%) | 0 (0%) | 0 (0%) | 0 (0%) | 0 (0%) | 2 (5.0%) | 4 (10.8%) | 4 (14.8%) |
| 5 | 0 (0%) | 0 (0%) | 0 (0%) | 0 (0%) | 0 (0%) | 0 (0%) | 1 (2.5%) | 1 (2.7%) | 0 (0%) |
| Missing | 62 (88.6%) | 54 (91.5%) | 29 (85.3%) | 0 (0%) | 1 (9.1%) | 0 (0%) | 0 (0%) | 0 (0%) | 1 (3.7%) |
| **ePAL** |  |  |  |  |  |  |  |  |  |
| Mean (SD) | 13.9 6.04) | 14.1 (6.25) | 14.6 (5.17) | 102 (59.1) | 104 (64.3) | 117 (82.2) | 180 (96.8) | 170 (94.6) | 176 (102) |
| Median [Min, Max] | 13.0  [5.0, 43.0] | 13.0  [5.0, 43.0] | 13.0  [5.0, 35.0] | 85.0  [55.0, 276] | 85.0  [55.0, 276] | 86.5  [55.0, 276] | 145  [67.0, 501] | 145  [67.0, 501] | 148  [80.0, 501] |
| Missing | 1 (1.4%) | 1 (1.7%) | 0 (0%) | 0 (0%) | 0 (0%) | 0 (0%) | 1 (2.5%) | 1 (2.7%) | 1 (3.7%) |
| **Cerebral WM FA** |  |  |  |  |  |  |  |  |  |
| Mean (SD) | 0.415 (0.0179) | 0.417 (0.0194) | 0.418 (0.0180) | 0.394 (0.0195) | 0.398 (0.0247) | 0.400 (0.0193) | 0.370 (0.0206) | 0.371 (0.0239) | 0.368 (0.0244) |
| Median [Min, Max] | 0.413 [0.37, 0.46] | 0.414 [0.376, 0.467] | 0.417 [0.375, 0.455] | 0.400 [0.353, 0.425] | 0.394 [0.362, 0.439] | 0.409 [0.375, 0.419] | 0.369 [0.332, 0.419] | 0.368 [0.332, 0.432] | 0.368 [0.330, 0.431] |
| Missing | 2 (2.9%) | 2 (3.4%) | 5 (14.7%) | 0 (0%) | 0 (0%) | 0 (0%) | 5 (12.5%) | 4 (10.8%) | 5 (18.5%) |

# Online references

1. Schielzeth H, Dingemanse NJ, Nakagawa S, et al. Robustness of linear mixed-effects models to violations of distributional assumptions. *Methods in Ecology and Evolution.* 2020;11(9):1141-1152.

2. Strang KH, Goodwin MS, Riffe C, et al. Generation and characterization of new monoclonal antibodies targeting the PHF1 and AT8 epitopes on human tau. *Acta Neuropathol Commun.* 2017;5(1):58.
